# 2-Rotund norms for unconditional and symmetric sequence spaces

**DOI:** 10.1007/s43037-024-00379-1

**Published:** 2024-09-14

**Authors:** Stephen Dilworth, Denka Kutzarova, Pavlos Motakis

**Affiliations:** 1 Department of Mathematics, University of South Carolina, Columbia, SC 29208 USA; 2 Department of Mathematics, University of Illinois Urbana-Champaign, Urbana, IL 61807 USA; 3 Institute of Mathematics and Informatics, Bulgarian Academy of Sciences, Sofia, Bulgaria; 4 Department of Mathematics and Statistics, York University, 4700 Keele Street, Toronto, ON M3J 1P3 Canada

**Keywords:** 2-Rotundity, Renorming, 2*R* norm, Unconditional basis, Symmetric basis, Reflexivity, 46B20, 46B10, 46B46, 46B70

## Abstract

A reflexive Banach space with an unconditional basis admits an equivalent 1-unconditional 2*R* norm and embeds into a reflexive space with a 1-symmetric 2*R* norm. Partial results on 1-symmetric 2*R* renormings of spaces with a symmetric basis are obtained.

## Introduction

The notions of 2-rotund and weakly 2-rotund norms were introduced by Milman [22] and are defined as follows.

### Definition 1.1

Let *X* be a Banach space. We say that a norm $\Vert \cdot \Vert$ on *X* is 2-rotund (2*R*) (resp.  weakly 2-rotund (*W*2*R*)) if for every $(x_n) \subset X$ such that $\Vert x_n\Vert \le 1$ ($n \ge 1$) and

$$\begin{aligned} \lim _{m,n \rightarrow \infty } \Vert x_m + x_n\Vert = 2, \end{aligned}$$

there exists $x \in X$ such that $x = \lim _{n \rightarrow \infty } x_n$ strongly (resp.  weakly).

Note that a *W*2*R* norm is strictly convex. It follows from a characterization of reflexivity due to James [17] that if *X* admits an equivalent *W*2*R* norm then *X* is reflexive. Hájek and Johanis [15] proved the converse: every reflexive Banach space admits an equivalent *W*2*R* norm. Odell and Schlumprecht [23] proved that every separable reflexive Banach space *X* admits an equivalent 2*R* norm (cf. [13]). However, it is an open question whether every reflexive Banach space admits an equivalent 2*R* norm (cf. [15, p. 72]).

One motivation for the present article is the following result of Figiel and Johnson which combines Theorem 3.1 and Remark 3.2 of [12]. Only the unconditional case is stated explicitly in [12], but the argument for the unconditional case also proves the symmetric case.

### Theorem A

Let *X* be a superreflexive Banach space with an unconditional (respectively, symmetric) basis $(e_n)_{n=1}^\infty$. Then *X* admits an equivalent uniformly convex norm for which $(e_n)_{n=1}^\infty$ is 1-unconditional (respectively, 1-symmetric).

Enflo [11] showed that a space is superreflexive if and only if it admits an equivalent uniformly convex norm. By the theorem of Odell and Schlumprecht above a separable space is reflexive if and only if it admits an equivalent 2*R* norm. Therefore it is natural to ask whether the analogue of Theorem A holds for 2*R* renormings of separable reflexive spaces.

In Sect. 3 we prove the analogous result in the unconditional case: a reflexive space with an unconditional basis admits a 1-unconditional 2*R* norm. For the symmetric case, however, we have only partial results. In particular, the following question is open.

### Question 1.2

Let *X* be a reflexive Banach space with a symmetric basis $(e_n)_{n=1}^\infty$. Does *X* admit an equivalent 2*R* norm for which $(e_n)_{n=1}^\infty$ is 1-symmetric?

We show that the answer is positive if the lower Boyd index $p_X$ of *X* satisfies $p_X>1$. We also prove that if *X* is a reflexive space with an unconditional basis then *X* is isomorphic to a 1-complemented subspace of a space with a 2*R* norm and a 1-symmetric basis. This is a refinement of a theorem of Szankowski [24]. A similar argument proves that the non-superreflexive space with a symmetric basis which does not contain $c_0$ or $\ell _p$ constructed in [12] and the space with a unique symmetric basic sequence (not equivalent to the unit vector basis of $c_0$ or $\ell _p$) constructed by Altshuler [1] both admit an equivalent 2*R* norm for which the basis is 1-symmetric.

A second motivation is the open question whether nonseparable reflexive spaces admit a 2*R* norm. To attack this problem it is natural to examine specific classes of nonseparable reflexive spaces for potential counterexamples or positive results. In [9] partial positive results were obtained for nonseparable generalized Baernstein spaces. Another natural class to examine is the class of spaces with an uncountable symmetric basis.

### Question 1.3

Suppose *X* is a reflexive space with an uncountable symmetric basis $(e_\gamma )_{\gamma \in \Gamma }$. Does *X* admit an equivalent (not necessarily 1-symmetric) 2*R* norm?

A positive answer to Quesion 1.2 would imply a positive answer to Question 1.3. In fact, it would imply that *X* admits a 1-symmetric 2*R* norm.

In the final section we show that $L_\infty [0,1]$ admits an equivalent rearrangement-invariant norm which restricts to a *W*2*R* norm on every reflexive subspace.

Finally, let us mention that related results are proved in [14, 16]. In [14] it is proved that a uniformly smooth (resp. uniformly convex) space with a Schauder basis admits a uniformly smooth (resp. uniformly convex) renorming for which the basis is monotone, while in [16] the spaces with a symmetric basis which admit equivalent symmetric norms that are Gâteaux differentiable or uniformly rotund in every direction are characterized.

## Preliminary results

We shall use the following characterization of 2-rotundity (see e.g., [8, II.6.4] or [15]): $\Vert \cdot \Vert$ is a 2*R* norm on *X* if for all $(x_n)_{n=1}^\infty \subset X$ such that

2.1 $$\begin{aligned} \lim _{m,n \rightarrow \infty } [\Vert x_m + x_n\Vert ^2 - 2(\Vert x_m\Vert ^2 + \Vert x_n\Vert ^2)] = 0, \end{aligned}$$

there exists $x \in X$ such that $x=\lim _{n \rightarrow \infty } x_n$ strongly.

Day [7] introduced the norm $\Vert \cdot \Vert _{\text {Day}}$ on $c_0$ defined by

$$\begin{aligned} \Vert (a_n)_{n=1}^\infty \Vert _{\text {Day}} = \left( \sum _{n=1}^\infty 4^{-n} a_n^{*2}\right) ^{1/2}, \end{aligned}$$

where $(a_n^*)_{n=1}^\infty$ is the non-increasing rearrangement of $(|a_n|)_{n=1}^\infty$. Let $(Y,\Vert \cdot \Vert )$ be a reflexive Banach space with normalized basis $(e_n)_{n=1}^\infty$. We define an equivalent norm on *Y* thus:

2.2 $$\begin{aligned} \left| \left| \left| \sum _{n=1}^\infty a_n e_n \ \right| \right| \right| =\left( \left\| \sum _{n=1}^\infty a_ne_n\right\| ^2 + \Vert (a_n)_{n=1}^\infty \Vert _{\text {Day}}^2\right) ^{1/2}. \end{aligned}$$

We will use the following result of Hájek and Johanis. It is a consequence of Theorem 3 and Corollary 4 of [15] and the reflexivity of *Y*. (Here $\Vert \sum _{n=1}^\infty a_n e_n\Vert _\infty = \sup _{n \ge 1} |a_n|$ as usual.)

### Theorem B

Suppose $$(y_n)_{n=1}^\infty \subset Y$$ satisfies

2.3 $$\begin{aligned} \lim _{m,n \rightarrow \infty }[ |\!|\!|y_n + y_m |\!|\!|^2- 2(|\!|\!|y_n|\!|\!|^2 + |\!|\!|y_m|\!|\!|^2) ]= 0. \end{aligned}$$

Then there exists $y \in Y$ such that

$$\begin{aligned} y_n\rightarrow y\quad \hbox { weakly as}\ n \rightarrow \infty \end{aligned}$$

and

$$\begin{aligned} \lim _{n \rightarrow \infty } \Vert y_n - y\Vert _\infty = 0. \end{aligned}$$

For $K \ge 1$, a basis $(e_n)_{n=1}^\infty$ is *K*-unconditional if

$$\begin{aligned} \left\| \sum _{n=1}^\infty \pm a_n e_n\right\| \le K\left\| \sum _{n=1}^\infty a_n e_n\right\| \end{aligned}$$

for all scalars $(a_n)_{n=1}^\infty$ and all choices of signs. The basis is *K*-symmetric if

$$\begin{aligned} \left\| \sum _{n=1}^\infty \pm a_{\sigma (n)} e_n\right\| \le K\left\| \sum _{n=1}^\infty a_n e_n\right\| \end{aligned}$$

for all scalars $(a_n)_{n=1}^\infty$, all choices of signs, and all permutations $\sigma :\mathbb {N} \rightarrow \mathbb {N}$.

We refer the reader to [20] for other unexplained Banach space notation and terminology.

## 1-Unconditional bases

### Theorem 3.1

Suppose that *X* has an unconditional basis. Then *X* admits an equivalent 1-unconditional norm $|\!|\!|\cdot |\!|\!|$ such that if $(x_n)_{n=1}^\infty \subset X$ is relatively weakly compact and satisifies (2.1), then $(x_n)$ converges strongly. In particular, if *X* is reflexive, then $|\!|\!|\cdot |\!|\!|$ is 2*R* and 1-unconditional.

### Proof

The proof closely follows [23, Main Theorem]. So it suffices to indicate how to adapt [23, Main Theorem] to produce a 1-unconditional basis as well as a 2*R* norm.

Let $(e_n)_{n=1}^\infty$ be a semi-normalized unconditional basis for *X* and let $\Vert \cdot \Vert$ denote any equivalent norm on *X* which is strictly convex and for which $(e_n)_{n=1}^\infty$ is 1-unconditional. To see that such a norm exists, let $|\cdot |$ be any equivalent norm on *X*. Let

$$\begin{aligned} \left\| \sum _{n=1}^\infty a_n e_n\right\| := \sup \left| \sum _{n=1}^\infty \pm a_n e_n\right| + \left( \sum _{n=1}^\infty 2^{-4n} a_n^2\right) ^{1/2}, \end{aligned}$$

where the supremum is taken over all choices of signs. Then $\Vert \cdot \Vert$ is strictly convex and $(e_n)_{n=1}^\infty$ is a 1-unconditional basis for $(X,\Vert \cdot \Vert )$. For $x \in X$, following [23, p. 148], define an equivalent norm $\Vert \cdot \Vert _x$ thus:

$$\begin{aligned} \Vert y\Vert _x:= \Vert \Vert y\Vert x + y\Vert + \Vert \Vert y\Vert x - y\Vert \quad (y \in X). \end{aligned}$$

Then [23, Lemma 2.1]

3.1 $$\begin{aligned} 2\Vert y\Vert \le \Vert y\Vert _x \le (2 + 2\Vert x\Vert )\Vert y\Vert .\end{aligned}$$

Let *C* be the countable vector space over $\mathbb {Q}$ defined by

$$\begin{aligned} C:= \left\{ \sum a_n e_n :(a_n) \in c_{00}, a_n \in \mathbb {Q}, n \ge 1\right\} . \end{aligned}$$

Let us say that $c = \sum a_ne_n \in C$ and $d = \sum b_ne_n \in C$ are absolutely equivalent if $|a_n| =|b_n|$ for all $n\ge 1$. Note that absolute equivalence is an equivalence relation on *C* and that the equivalence classes are finite. Let $\mathcal {A}$ be the collection of equivalence classes. For all $A \in \mathcal {A}$ and for all absolutely equivalent $y,z \in C$, by 1 unconditionality of $\Vert \cdot \Vert$ and absolute equivalence of *y* and *z*, we have

3.2 $$\begin{aligned} \begin{aligned} \sum _{c \in A} \Vert y\Vert _c&= \sum _{c \in A}( \Vert \Vert y\Vert c + y\Vert + \Vert \Vert y\Vert c - y\Vert )\\&= \sum _{c \in A}( \Vert \Vert z\Vert c + z\Vert + \Vert \Vert z\Vert c - z\Vert )\\&=\sum _{c \in A} \Vert z\Vert _c. \end{aligned}\end{aligned}$$

Choose $p_A>0$ ($A \in \mathcal {A}$) such that $\sum _{A \in \mathcal {A} }p_A(1 + \sum _{c \in A} \Vert c\Vert ) < \infty$. Define a norm $|\!|\!|\cdot |\!|\!|$ on *X* as follows:

$$\begin{aligned} |\!|\!|x|\!|\!| = \sum _{A \in \mathcal {A}} p_A \sum _{c \in A} \Vert x\Vert _c. \end{aligned}$$

It follows from (3.1) that $|\!|\!|\cdot |\!|\!|$ is an equivalent norm. Note that $|\!|\!|\cdot |\!|\!|$ is strictly convex since $\Vert \cdot \Vert _0 = 2\Vert \cdot \Vert$ which is strictly convex. Suppose $y,z \in C$ are absolutely equivalent. Then (3.2) implies that

$$\begin{aligned} |\!|\!|y|\!|\!| = \sum _{A \in \mathcal {A}} p_A \sum _{c \in A} \Vert y\Vert _c = \sum _{A \in \mathcal {A}} p_A \sum _{c \in A} \Vert z\Vert _c = |\!|\!|z|\!|\!|. \end{aligned}$$

Since *C* is dense in *X*, it follows that $(e_n)$ is a 1-unconditional basis for $(X, |\!|\!|\cdot |\!|\!|)$. The proof of [23, Main Theorem], especially Lemmas 2.2(a), 2.3(a), and 2.4, now shows that $\Vert \cdot \Vert _M$ is 2*R*. $\square$

### Remark 3.2

We are unable to adapt the proof of Theorem 3.1 to the case of a symmetric basis. The natural approach would be to say that vectors from *C* are equivalent if their coefficient sequences are permuted. However, in this case the equivalence classes are infinite, so the proof does not go through.

Lindenstrauss [19] proved that every space *X* with an unconditional basis is isomorphic to a complemented subspace of a space *Y* with a symmetric basis. Subsequently, Szankowski [24] proved that if *X* is reflexive then *Y* can be chosen to be reflexive and Davis [5] proved that if *X* is superreflexive then *Y* can be chosen to be superreflexive. Davis’s method also proves the reflexive case. As an application of Theorem 3.1 we use Davis’s method to prove the following refinement of Szankowski’s result.

### Theorem 3.3

Suppose that *X* is reflexive and has an unconditional basis. Then *X* is isomorphic to a 1-complemented subspace of a space with a 1-symmetric basis and a 2*R* norm. Moreover, that subspace has a 1-unconditional basis.

We recall the presentation of Davis’s approach in [20, p. 125]. Let $(E, \Vert \cdot \Vert )$ and $(F,\Vert \cdot \Vert )$ be two Banach sequence spaces such that $(e_n)_{n=1}^\infty$ is a 1-symmetric basis for both *E* and *F*. We assume also that $\Vert x\Vert _E \le \Vert x\Vert _F$ for all $x \in F$ and that

$$\begin{aligned} \lim _{n \rightarrow \infty } \frac{\Vert \sum _{i=1}^n e_i\Vert _E}{\Vert \sum _{i=1}^n e_i\Vert _F} = 0. \end{aligned}$$

For each $m \ge 1$, define a 1-symmetric norm $\Vert \cdot \Vert _m$ on *E* as follows:

$$\begin{aligned} \Vert x\Vert _m = \inf \left\{ (\Vert y\Vert _E^2 + \Vert z\Vert _F^2)^{1/2} :x = \frac{1}{m} y + mz, y \in E, z \in F\right\} . \end{aligned}$$

Then [20, p. 125]

3.3 $$\begin{aligned} \frac{1}{m} \Vert x\Vert _m \le \Vert x\Vert _E \le 2m \Vert x\Vert _m \quad (x \in E). \end{aligned}$$

Hence $\Vert \cdot \Vert _m$ is equivalent to $\Vert \cdot \Vert _E$.

Now suppose that *X* is a Banach space with a normalized 1-unconditional basis $(f_n)_{n=1}^\infty$. For every strictly increasing sequence $(m_n)_{n=1}^\infty$ such that $\sum _{n=1}^\infty 1/m_n < \infty$, we define the space $Y:= Y(E,F,X, (m_n)_{n=1}^\infty )$ to be the collection of all $x \in E$ for which

3.4 $$\begin{aligned} \Vert x\Vert _Y:= \left\| \sum _{n=1}^\infty \Vert x\Vert _{m_n} f_n\right\| _X < \infty . \end{aligned}$$

Then $(e_n)$ is a 1-symmetric basis for *Y*. (The condition $\sum _{n=1}^\infty 1/m_n < \infty$ guarantees that *F* embeds continuously into *Y* and, in particular, that $(e_n)_{n=1}^\infty \subset Y$ [20, p. 126].)

### Theorem C

[20, Prop. 3.b.4] For every *E*, *F* and *X* as above there exists an increasing sequence of numbers $(m_n)_{n=1}^\infty$ with $\sum _{n=1}^\infty 1/m_n< \infty$ such that $Y = Y(E,F,X,(m_n)_{n=1}^\infty )$ contains a complemented subspace isomorphic to *X*.

The following lemma generalizes [20, Lemma 3.b.11].

### Lemma 3.4

Suppose that $E=c_0$ and $\sum _{n=1}^\infty 1/ m_n < \infty$. Let

$$\begin{aligned} u_n = \sum _{i = q_n +1}^{q_{n+1}} c_i e_i \quad (m \ge 1) \end{aligned}$$

be a normalized block basis (with respect to $(e_n)_{n=1}^\infty )$ in $Y(c_0,F,X,(m_n)_{n=1}^\infty )$ such that $\lim _{i \rightarrow \infty } c_i = 0$. Then some subsequence of $(u_n)_{n=1}^\infty$ is equivalent to a block basis of $(f_n)_{n=1}^\infty$ in *X*.

### Proof

Fix $N \ge 1$ and $n \ge 1$. It follows from (3.3) that

$$\begin{aligned} \sum _{i=1}^N \Vert u_n\Vert _{m_i} \le \left( \sum _{i=1}^N m_i\right) \Vert u_n\Vert _E = \left( \sum _{i=1}^N m_i\right) \max \{|c_i| :q_n+1 \le i \le q_{n+1}\}. \end{aligned}$$

Since $\lim _{i \rightarrow \infty } c_i = 0$, we can inductively define an increasing sequence of natural numbers $1=N_1< N_2<\cdots$ and a subsequence $(u_{n_k})_{k=1}^\infty$ such that for all $k \ge 1$

$$\begin{aligned} \left\| \sum _{i=1}^{N_k} \Vert u_{n_{k}}\right\| _{m_i} f_i\Vert _X + \left\| \sum _{N_{k+1}+1} ^\infty \right\| u_{n_{k}}\Vert _{m_i} f_i\Vert _X < 2^{-k-1}. \end{aligned}$$

It follows that the block basis $(u_{n_k})_{k=1}^\infty \subset Y$ is equivalent to the block basis

$$\begin{aligned} \left( \sum _{i = N_k+1}^{N_{k+1}} \Vert u_{n_k}\right\| _{m_i} f_i )_{k=1}^\infty \subset X. \end{aligned}$$

$$\square $$

The next lemma is more general than is needed for the proof of Theorem 3.3, but we believe that the additional generality may be of independent interest.

### Lemma 3.5

Suppose that $E=c_0$ and *X* is reflexive. If $\sum _{n=1}^\infty 1/m_n < \infty$ then $Y(c_0,F,X, (m_n)_{n=1}^\infty )$ is reflexive.

### Proof

Since *Y* has a symmetric (hence unconditional) basis it follows from a result of James [18] that *Y* is reflexive unless *Y* contains a subspace isomorphic to $c_0$ or $\ell _1$. We use the fact that every (infinite-dimensional) subspace of *Y* contains a further subspace isomorphic to a subspace of *X* or to a subspace of *E* (see [20, p. 127]). Since every subspace of $\ell _1$ contains a further subspace isomorphic to $\ell _1$, and since neither $c_0$ nor *X* contain a subspace isomorphic to $\ell _1$, it follows that *Y* does not contain a subspace isomorphic to $\ell _1$. Suppose, to obtain a contradiction, that *Y* contains a sequence equivalent to the unit vector basis of $c_0$. Since the unit vector basis of $c_0$ is weakly null, a standard gliding hump argument shows that $(e_n)_{n=1}^\infty$ admits a block basis

$$\begin{aligned} u_n= \sum _{i = q_n +1}^{q_{n+1}} c_i e_i \end{aligned}$$

equivalent to the unit vector basis of $c_0$. By the proof of [20, Lemma 3.b.3],

$$\begin{aligned} \max _{n\ge 1} \left\| \sum _{i=1}^n e_i\right\| _m \rightarrow \infty \quad \text {as } \, m \rightarrow \infty . \end{aligned}$$

So, using (3.4), $\Vert \sum _{i=1}^n e_i\Vert _Y \rightarrow \infty$ as $n \rightarrow \infty$. It follows from the unconditionality of $(e_n)_{n=1}^\infty$ and the fact that $\sup _{n\ge 1}\Vert \sum _{i=1}^n u_i\Vert _Y < \infty$ that $\lim _{i \rightarrow \infty } c_i =0$. By Lemma 3.4, some subsequence of $(u_n)_{n=1}^\infty$ is equivalent to a block basis of $(f_n)_{n=1}^\infty \subset X$. Hence $c_0$ is isomorphic to a subspace of *X*, which contradicts the reflexivity of *X*. $\square$

### Remark 3.6

Reflexivity of $Y(c_0, \ell _1,X,( 2^n)_{n=1}^\infty )$ was proved in [12] using results from [6].

### Proof

(Proof of Theorem 3.3) By Theorem 3.1, *X* has an equivalent 2*R* norm $\Vert \cdot \Vert _X$ for which $(f_n)_{n=1}^\infty$ is a 1-unconditional basis. Let $Y:=Y(c_0, F, X, (m_n)_{n=1}^\infty )$ be as in Theorem C. We equip *Y* with the equivalent norm defined by (2.2).

Suppose $(y_n)_{n=1}^\infty$ satisfies (2.3). Since *Y* is reflexive, by Theorem B there exists $y \in Y$ such that $y_n \rightarrow y$ weakly and $\Vert y_n - y\Vert _\infty \rightarrow 0$ as $n \rightarrow \infty$. Moreover, (2.3) implies that

3.5 $$\begin{aligned} \lim _{m,n \rightarrow \infty }[\Vert y_n + y_m \Vert _Y^2- 2(\Vert y_n\Vert _Y^2 + \Vert y_m\Vert _Y^2)] = 0. \end{aligned}$$

Let $x_n = \sum _{i=1}^\infty \Vert y_n\Vert _{m_i} f_i$ ($n \ge 1$). By definition of $\Vert \cdot \Vert _Y$, $\Vert x_n\Vert _X = \Vert y_n\Vert _Y$. Moreover, by 1-unconditionality of the basis $(f_n)_{n=1}^\infty$ of *X*,

$$\begin{aligned} \Vert x_n + x_k\Vert _X&= \left\| \sum _{i=1}^\infty (\Vert y_n\Vert _{m_i} + \Vert y_k\Vert _{m_i}) f_i\right\| _X\\&\ge \left\| \sum _{i=1}^\infty \Vert y_n\ + y_k\Vert _{m_i}\ f_i\right\| _X\\&= \Vert y_n + y_k\Vert _Y. \end{aligned}$$

Hence (3.5) implies that

$$\begin{aligned} \lim _{m,n \rightarrow \infty }[\Vert x_n + x_m \Vert _X^2- 2(\Vert x_n\Vert _X^2 + \Vert x_m\Vert _X^2)] = 0. \end{aligned}$$

Since $\Vert \cdot \Vert _X$ is 2*R*, it follows that $(x_n)_{n=1}^\infty$ is a Cauchy sequence in *X*. Hence, given $\varepsilon >0$, there exists $N_1\ge 1$ such that

$$\begin{aligned} \left\| \sum _{i=N_1+1}^\infty \Vert y_n\Vert _{m_i} f_i\right\| _X < \frac{\varepsilon }{4} \quad (n \ge 1). \end{aligned}$$

Hence

3.6 $$\begin{aligned} \left\| \sum _{i=N_1+1}^\infty \Vert y_n-y_k\Vert _{m_i} f_i\right\| _X < \frac{\varepsilon }{2} \quad (n,k \ge 1). \end{aligned}$$

Recall that, by (3.3), $\Vert \cdot \Vert _m$ is equivalent to $\Vert \cdot \Vert _\infty$ for all $m \ge 1$. Since $\lim _{n \rightarrow \infty } \Vert y_n - y\Vert _\infty = 0$, it follows that $(y_n)_{n=1}^\infty$ is a Cauchy sequence in $\Vert \cdot \Vert _m$ for all $m \ge 1$. Hence there exists $N_2 \ge 1$ such that

3.7 $$\begin{aligned} \left\| \sum _{i=1}^{N_1} \Vert y_n - y_k\Vert _{m_i} f_i\right\| _X < \frac{\varepsilon }{2} \quad (n,k \ge N_2). \end{aligned}$$

Combining (3.6) and (3.7), we have $\Vert y_n - y_k\Vert _Y < \varepsilon$ for all $n,k \ge N_2$. So $(y_n)_{n=1}^\infty$ is a Cauchy sequence in *Y* and hence $\lim _{n \rightarrow \infty } \Vert y_n - y\Vert _Y = 0$.

The proof of Theorem C shows that *X* is isomorphic to the closed linear span *Z* of disjointly supported constant coefficient vectors in *Y*. Hence *Z* has a 1-unconditional basis and is the range of an averaging projection on *Y*. So *Z* is 1-complemented in *Y*. $\square$

Let *T* be the space introduced in [12] (the dual of the space that does not contain $c_0$ or $\ell _p$ constructed by Tsirelson [4]). It was proved in [12] that $Y(c_0,\ell _1,T, (2^n)_{n=1}^\infty )$ does not contain a subspace isomorphic to $c_0$ or $\ell _p$.

Let $d_{w,1}$ be the Lorentz sequence space corresponding to the weight sequence $w = (1/n)$. The norm in $d_{w,1}$ is given by

$$\begin{aligned} \left\| \sum _{n=1}^\infty a_n e_n\right\| = \sum _{n=1}^\infty \frac{ a_n^*}{n}. \end{aligned}$$

It was proved by Altshuler [1] that $Y(c_0, d_{w,1},T,(2^n)_{n=1}^\infty )$ has a unique symmetric basic sequence which, moreover, is not equivalent to the unit vector basis of $c_0$ or $\ell _p$.

The proof of Theorem 3.3 also establishes the following result.

### Theorem 3.7

The spaces $Y(c_0,\ell _1,T, (2^n)_{n=1}^\infty )$ of [12] and $Y(c_0, d_{w,1},T,(2^n)_{n=1}^\infty )$ of [1] both have equivalent 2*R* norms with a 1-symmetric basis.

## 1-Symmetric bases

In this section $(X, \Vert \cdot \Vert )$ denotes a reflexive Banach space with a symmetric basis $(e_n)_{n=1}^\infty$.

Let us recall the definition of the lower Boyd index [3] $p_X$ of *X* (cf. [21, p. 130]). For $m \in \mathbb {N}$, the linear operator $D_m :X \rightarrow X$ is defined by

$$\begin{aligned} D_m\left( \sum _{n=1}^\infty a_n e_n\right) = \sum _{n=1}^\infty a_n \left( \sum _{j=(n-1)m+1}^{nm} e_j\right) . \end{aligned}$$

The lower Boyd index $p_X$ is defined by

4.1 $$\begin{aligned} p_X = \sup _{m \ge 2} \frac{\log m}{\log \Vert D_m\Vert } = \lim _{m \rightarrow \infty } \frac{\log m}{\log \Vert D_m\Vert }. \end{aligned}$$

The following main result of this section is an immediate consequence of Theorem 4.7 proved below.

### Theorem 4.1

Suppose that *X* is a reflexive Banach space with a symmetric basis such that $p_X >1$. Then *X* admits a 1-symmetric 2*R* norm.

[21, Prop. 2.b.7], which characterizes when $p_X >1$, yields a geometrical formulation of Theorem 4.1.

### Corollary 4.2

Suppose that *X* is a reflexive Banach space with a symmetric basis which does not admit uniformly isomorphic copies of $\ell _1^n$ spanned by disjointly supported vectors with the same distribution. Then *X* admits a 1-symmetric 2*R* norm.

For $x = \sum _{i=1}^\infty x(i)e_i \in X$, define the formal series

$$\begin{aligned} \widehat{x}: = \sum _{n=1}^\infty \left( \frac{1}{n} \sum _{i=1}^n x^*(i)\right) e_n. \end{aligned}$$

We prove the following lemma for the sake of completeness. More general results in the setting of rearrangement-invariant function spaces rather than symmetric sequence spaces are proved in [2, Theorem 5.15].

### Lemma 4.3

Suppose that $p_X >1$. Then there exists a constant $c > 0$ such that

$$\begin{aligned} \Vert \widehat{x}\Vert \le c \Vert x\Vert \quad (x \in X). \end{aligned}$$

### Proof

We may assume that $(e_n)_{n=1}^\infty$ is a 1-symmetric basis of *X*. Let $1< p < p_X$. It follows from (4.1) that there exists $A>0$ such that $\Vert D_m\Vert \le A m^{1/p}$ for all $m \ge 1$. Consider $x = \sum _{n=1}^\infty x(n) e_n \in X$, where $(x(n))_{n=1}^\infty$ is a nonnegative decreasing sequence. Define $f :(0,\infty ) \rightarrow (0,\infty )$ by $f(t) = x(n)$ for $n\ge 1$ and $n-1 < t \le n$. Then

$$\begin{aligned} \widehat{x} = \sum _{n=1}^\infty \left( \int _0^1 f(tn) \, dt\right) e_n = \int _0^1 \left( \sum _{n=1}^\infty f(tn)e_n\right) \, dt \end{aligned}$$

Hence

$$\begin{aligned} \Vert \widehat{x}\Vert&\le \int _0^1 \left\| \sum _{n=1}^\infty f(tn)e_n\right\| \, dt\\&\le \sum _{m=1}^\infty 2^{-m} \left\| \sum _{n=1}^\infty f(2^{-m}n)e_n\right\| \end{aligned}$$

(by 1-unconditionality)

$$\begin{aligned}&= \sum _{m=1}^\infty 2^{-m} \Vert D_{2^m}(x)\Vert \\&\le \sum _{m=1}^\infty 2^{-m} (A 2^{m/p}) \Vert x\Vert \\&= \frac{A}{ 2^{1-1/p }-1}\Vert x\Vert . \end{aligned}$$

$$\square $$

Henceforth, we suppose that $p_X>1$, and, using Theorem 3.1, that $\Vert \cdot \Vert$ is 2*R*, and that $(e_n)_{n=1}^\infty$ is a symmetric 1-unconditional basis for $\Vert \cdot \Vert$. Suppose that $(e_n)_{n=1}^\infty$ is *K*-symmetric for $\Vert \cdot \Vert$. Define a quasi-norm $|\!|\!|\cdot |\!|\!|$ as follows:

4.2 $$\begin{aligned} |\!|\!|x |\!|\!| = (\Vert \widehat{x}\Vert ^2 + \Vert (x(n))_{n=1}^\infty \Vert _{\text {Day}}^2)^{1/2} \quad \left( x = \sum _{n=1}^\infty x(n) e_n \in X\right) . \end{aligned}$$

### Lemma 4.4

$|\!|\!|\cdot |\!|\!|$ is a 1-symmetric equivalent norm on *X*.

### Proof

Clearly, $|\!|\!|\cdot |\!|\!|$ is a 1-symmetric quasi-norm since $\widehat{x}$ depends only on $(x^*(n))_{n=1}^\infty$ and $\Vert \cdot \Vert _{\text {Day}}$ is 1-symmetric. For $x \in X$,

$$\begin{aligned} \frac{1}{K} \Vert x\Vert&= \frac{1}{K} \left\| \sum _{n=1}^\infty x(n) e_n\right\| \\&\le \left\| \sum _{n=1}^\infty x^*(n) e_n\right\| \end{aligned}$$

(since $(e_n)_{n=1}^\infty$ is a *K*-symmetric basis)

$$\begin{aligned}&\le \Vert \hat{x}\Vert \end{aligned}$$

(since $x^*(n) \le \frac{1}{n} \sum _{i=1}^n x^*(i)$ and $(e_n)_{n=1}^\infty$ is a 1-unconditional basis)

$$\begin{aligned}&\le c \Vert x\Vert . \end{aligned}$$

Since $\Vert \cdot \Vert _{\text {Day}}$ is equivalent to $\Vert \cdot \Vert _\infty$, it follows that $\Vert \cdot \Vert$ and $|\!|\!|\cdot |\!|\!|$ are equivalent quasi-norms. It remains to prove that $|\!|\!|\cdot |\!|\!|$ is in fact a norm, i.e., that $|\!|\!|\cdot |\!|\!|$ satisfies the triangle inequality. It suffices to show that $x \mapsto \Vert \widehat{x}\Vert$ satisfies the triangle inequality. Let $x,y \in X$. Note that

4.3 $$\begin{aligned} \widehat{(x+y)}(n) \le \widehat{x}(n)+ \widehat{y}(n) \quad (n \in \mathbb {N}). \end{aligned}$$

Since $(e_n)_{n=1}^\infty$ is a 1-unconditional basis, it follows that

4.4 $$\begin{aligned} \Vert \widehat{x+y}\Vert \le \Vert \widehat{x} + \widehat{y}\Vert \le \Vert \widehat{x}\Vert +\Vert \widehat{y}\Vert . \end{aligned}$$

Hence $x \mapsto \Vert \widehat{x}\Vert$ and $\Vert \cdot \Vert$ are equivalent norms on *X*.

$$\square $$

For $x \in X$ and $N,M \in \mathbb {N}$, define

$$\begin{aligned} x\cdot 1_{[N,M]}:= \sum _{n=N}^M x(n)e_n. \end{aligned}$$

### Lemma 4.5

For $$x,y \in X$$ and $$N \in \mathbb {N}$$,

$$\begin{aligned} | \Vert \widehat{x}\cdot 1_{[N,\infty )}\Vert - \Vert \widehat{y}\cdot 1_{[N, \infty )}\Vert | \le c \Vert x-y\Vert . \end{aligned}$$

### Proof

(4.3) yields

$$\begin{aligned} \Vert \widehat{x}\cdot 1_{[N,\infty )}\Vert \le \Vert \widehat{y}\cdot 1_{[N,\infty )}\Vert + \Vert (\widehat{x-y})\cdot 1_{[N,\infty )}\Vert . \end{aligned}$$

Hence

$$\begin{aligned} \Vert \widehat{x}\cdot 1_{[N,\infty )}\Vert - \Vert \widehat{y}\cdot 1_{[N,\infty )}\Vert \le \Vert (\widehat{x-y})\cdot 1_{[N,\infty )}\Vert \le \Vert (\widehat{x-y})\Vert \le c\Vert x-y\Vert . \end{aligned}$$

Interchanging *x* and *y* gives the result. $\square$

### Lemma 4.6

Suppose that $x \in X$, $y_n \in X$ ($n \ge 1$), $\Vert y_n\Vert \ge \delta >0$, $\lim _{n \rightarrow \infty } \Vert y_n\Vert _\infty =0$ and $\min ({\text {supp}} (y_n)) \rightarrow \infty$. Then, for all $N \ge 1$,

$$\begin{aligned} \liminf _{n \rightarrow \infty } \Vert (\widehat{x+y_n})\cdot 1_{[N, \infty )} \Vert \ge \frac{\delta }{K}. \end{aligned}$$

### Proof

Let $\alpha >0$. Choose $N_1 \in \mathbb {N}$ so that $x^{\prime }:= x 1_{[1,N_1]}$ satisfies $\Vert x - x^{\prime }\Vert < \alpha$. Then, for all sufficiently large *n*, we have

$$\begin{aligned} \min ( {\text {supp}}(y_n)) > N_1 \ge \max ({\text {supp}}(x^{\prime }). \end{aligned}$$

Hence, for all sufficiently large *n* and for all $i \ge 1$,

$$\begin{aligned} (\widehat{x^{\prime } + y_n})(i) \ge y_n^*(i). \end{aligned}$$

So for all $N \ge 1$ and for all sufficiently large *n*,

$$\begin{aligned} \Vert (\widehat{x^\prime + y_n)} 1_{[N,\infty )}\Vert&\ge \left\| \sum _{i = N+1}^\infty y_n^*(i) e_i\right\| \\&\ge \left\| \sum _{i=1}^\infty y_n^*(i) e_i\right\| - N\Vert y_n\Vert _\infty \\&\ge \frac{1}{K} \Vert y_n\Vert -N \Vert y_n\Vert _\infty \\  &\ge \frac{\delta }{K} - N\Vert y_n\Vert _\infty . \end{aligned}$$

Hence, by Lemma 4.5, for all $N \ge 1$ and for all sufficiently large *n*,

$$\begin{aligned} \Vert (\widehat{x + y_n})\cdot 1_{[N,\infty )}\Vert&\ge \Vert (\widehat{x ^\prime + y_n})\cdot 1_{[N,\infty )}\Vert - c\Vert x - x^\prime \Vert \\  &\ge \frac{\delta }{K} - N\Vert y_n\Vert _\infty -c\alpha . \end{aligned}$$

Since $\lim _{n \rightarrow \infty } \Vert y_n\Vert _\infty =0$ and $\alpha >0$ is arbitrary the result follows. $\square$

### Theorem 4.7

$|\!|\!|\cdot |\!|\!|$ is a 1-symmetric 2*R* equivalent norm on *X*.

### Proof

Let us summarize the relevant progress we have made so far in this section. We used Theorem 3.1 to equip *X* with an equivalent 2R norm $\Vert \cdot \Vert$ that is 1-unconditional but not necessarily 1-symmetric (see the paragraph before Lemma 4.4). We then defined the equivalent norm $|\!|\!|\cdot |\!|\!|$, which we have shown in Lemma 4.4 to be 1-symmetric. It remains to prove that it is 2R. Suppose $(x_n)_{n=1}^\infty \subset X$ satisfies

4.5 $$\begin{aligned} \lim _{m,n \rightarrow \infty }[ |\!|\!|x_n + x_m |\!|\!|^2- 2(|\!|\!|x_n|\!|\!|^2 + |\!|\!|x_m|\!|\!|^2) ]= 0. \end{aligned}$$

By Theorem B, $(x_n)_{n=1}^\infty$ converges weakly to some $x \in X$ and $\lim _{n \rightarrow \infty } \Vert x_n - x\Vert _\infty = 0$. Let $x_n = x + y_n$ and suppose that $(y_n)_{n=1}^\infty$ does not converge to zero in norm. Since $\lim _{n \rightarrow \infty }\Vert y_n\Vert _\infty = 0$, a gliding hump and an approximation argument show, after passing to a subsequence and relabelling, that without loss of generality each $y_n$ has finite support, that $(y_n)_{n=1}^\infty$ is a block basis with respect to $(e_n)_{n=1}^\infty$, and hence $\min ( {\text {supp}}(y_n)) \rightarrow \infty$ as $n \rightarrow \infty$, and that $\Vert y_n\Vert> \delta >0$ ($n \ge 1$).

It follows from (4.5) and the definition of $|\!|\!|\cdot |\!|\!|$ in (4.2) that

$$\begin{aligned} \lim _{m,n \rightarrow \infty } \Vert \widehat{x_n + x_m} \Vert ^2- 2(\Vert \widehat{ x_n} \Vert ^2 + \Vert \widehat{x_m}\Vert ^2) = 0. \end{aligned}$$

Note that $\Vert (\widehat{x_n + x_m}) \Vert \le \Vert \widehat{x_n} +\widehat{ x_m} \Vert$ by (4.4). Hence

$$\begin{aligned} \lim _{m,n \rightarrow \infty } \Vert \widehat{x_n} + \widehat{x_m} \Vert ^2- 2(\Vert \widehat{ x_n} \Vert ^2 + \Vert \widehat{x_m}\Vert ^2) = 0. \end{aligned}$$

Since $\Vert \cdot \Vert$ is a 2*R* equivalent norm on *X*, it follows that $(\widehat{x_n})_{n=1}^\infty$ converges strongly in *X*. By Lemma 4.6, for all $N \ge 1$,

$$\begin{aligned} \liminf _{m \rightarrow \infty } \Vert \widehat{x_m}\cdot 1_{[N,\infty )} \Vert = \liminf _{m \rightarrow \infty }\Vert (\widehat{x+y_m})\cdot 1_{[N,\infty )}\Vert \ge \frac{\delta }{K}, \end{aligned}$$

which contradicts the fact that $(\widehat{x_n})_{n=1}^\infty$ is a Cauchy sequence in *X*. $\square$

## Symmetric renormings of $\ell _\infty$ and $L_\infty$

A symmetric renorming of $\ell _\infty$ [16] is an equivalent norm $\Vert \cdot \Vert$ on $\ell _\infty$ such that

$$\begin{aligned} \Vert (a_n)_{n=1}^\infty \Vert = \Vert (a_{\sigma (n)})_{n=1}^\infty \Vert \quad ((a_n)_{n=1}^\infty \in \ell _\infty ) \end{aligned}$$

for all permutations $\sigma$ of $\mathbb {N}$. It was proved in [16, Proposition 5] that for every symmetric renorming $\Vert \cdot \Vert$, $(\ell _\infty ,\Vert \cdot \Vert )$ contains a subspace isometric to $(\ell _\infty , \Vert \cdot \Vert _\infty )$. For the sake of completeness we include an elementary proof that avoids uncountable cardinals.

### Theorem 5.1

Let $\Vert \cdot \Vert$ be a 1-symmetric norm on $\ell _\infty$. Then there exists a subspace *Y* of $(\ell _\infty ,\Vert \cdot \Vert )$ that is isometrically isomorphic to $(\ell _\infty ,\Vert \cdot \Vert _\infty )$. In fact, there exists $\alpha >0$ such that, for all $y \in Y$, $\Vert y\Vert = \alpha \Vert y\Vert _\infty$.

### Proof

Let $\Vert \cdot \Vert$ be a 1-symmetric norm on $\ell _\infty$. We let $2\mathbb {N}$ denote the set of even positive integers and $\ell _\infty (2\mathbb {N})$ the subspace of $\ell _\infty$ comprising all *x* with $\textrm{supp}(x) = \{i\in \mathbb {N}:x(i)\ne 0\}\subset 2\mathbb {N}$. We will first show that $\Vert \cdot \Vert$ restricted on $\ell _\infty (2\mathbb {N})$ is 1-suppression unconditional, i.e., for every $x,y\in \ell _\infty (2\mathbb {N})$ such that, for all $i\in \mathbb {N}$, $x(i) = y(i)$ or $y(i) = 0$, we have $\Vert x\Vert \ge \Vert y\Vert$. We verify this on the dense linear subspace of $\ell _\infty (2\mathbb N)$ consisting of all *x* that have the form

$$\begin{aligned}x = \sum _{i=1}^na_i\chi _{A_i},\end{aligned}$$

where $n\in \mathbb N$, $a_1,\ldots ,a_n$ are (not necessarily different) scalars, and $A_1,\ldots ,A_n$ are disjoint (finite or infinite) subsets of $2\mathbb N$. By symmetry, it suffices to show that, letting

$$\begin{aligned}y = \sum _{i=1}^{n-1}a_i\chi _{A_i},\end{aligned}$$

$\Vert x\Vert \ge \Vert y\Vert$. Fix an infinite $S\subset \mathbb N\setminus 2\mathbb N$. For each $N\in \mathbb N$, choose disjoint subsets $A_n^{1},\ldots ,A_n^{N}$ of *S* that are equinumerous to $A_n$, and, for $1\le j\le N$, let $x_N^{j} = y+a_n\chi _{A_n^j}$. By symmetry, $\Vert x_N^j\Vert = \Vert x\Vert$, and thus letting

$$\begin{aligned}y_N = \frac{1}{N}\sum _{j=1}^Nx_N^{j}\end{aligned}$$

we have $\Vert x\Vert \ge \Vert y_N\Vert$. At the same time, $\Vert y_N - y\Vert _\infty \rightarrow 0$, and thus by equivalence $\Vert y_N - y\Vert \rightarrow 0$, which yields $\Vert x\Vert \ge \Vert y\Vert$.

By scaling and symmetry, we may assume that, for all $n \in \mathbb {N}$, $\Vert e_n\Vert =1$ and thus, by 1-suppression unconditionality, for all $x \in \ell _\infty (2\mathbb N)$, $\Vert x\Vert \ge \Vert x\Vert _\infty$. Put

$$\begin{aligned} \alpha = \sup \{\Vert x\Vert :x \in \ell _\infty (2\mathbb {N}),\Vert x\Vert _\infty = 1\}. \end{aligned}$$

Then there exists $x_0 \in \ell _\infty (2\mathbb {N})$ such that $\Vert x_0\Vert _\infty = 1$ and $\Vert x_0\Vert = \alpha$. Indeed, by symmetry we can pick disjointly supported vectors $(x_n)_{n=1}^\infty$ in $\ell _\infty (2\mathbb {N})$ such that, for all $n \in \mathbb {N}$, $\Vert x_n\Vert _\infty = 1$ and $\Vert x_n\Vert \ge \alpha - 1/n$. This follows from the symmetry of $\Vert \cdot \Vert$ and the fact that any bijection between infinite subsets of $2 \mathbb {N}$ extends to a permutation of $\mathbb {N}$. Define $x_0 = \sum _{n=1}^\infty x_n$ pointwise. Then, $\Vert x_0\Vert _\infty = 1$ and thus $\Vert x_0\Vert \le \alpha$. By 1-suppression unconditionality, for all $n \in \mathbb {N}$, $\Vert x_0\Vert \ge \Vert x_n\Vert \ge \alpha -1/n$. So $\Vert x_0\Vert = \alpha$. Pick a disjointly supported sequence $(y_n)_{n=1}^\infty$ in $\ell _\infty (2\mathbb {N})$ so that each $y_n$ has the same distribution as $x_0$. Then, for all $(a_n) \in \ell _\infty$, $\sum _{n=1}^\infty a_n y_n$ (defined pointwise) satisfies $\Vert \sum _{n=1}^\infty a_n y_n\Vert = \alpha \Vert (a_n)\Vert _\infty .$ Hence $Y = \{\sum _{n=1}^\infty a_n y_n :(a_n) \in \ell _\infty \}$ has the required property. $\square$

On the other hand, by [15, Corollary 4] $\ell _\infty$ admits an equivalent norm which restricts to a *W*2*R* norm on reflexive subspaces. Clearly, every such norm is strictly convex and hence cannot be symmetric by Theorem 5.1.

Next we consider rearrangement-invariant renormings of $L_\infty [0,1]$. Curiously, we reach a rather different conclusion from the case of $\ell _\infty$.

We will apply the following result from [10].

### Theorem D

[10] There is an equivalent rearrangement-invariant (Orlicz) norm $|\!|\!|\cdot |\!|\!|$ on $L_1[0,1]$ satisfying the following restricted uniform convexity condition. Let $K \subset \{x \in L_1[0,1] :|\!|\!|x |\!|\!| \le 1\}$ be weakly compact. Then, given $\varepsilon >0$, there exists $\delta >0$ such that for all $x,y \in K$,

$$\begin{aligned} |\!|\!|x + y |\!|\!| > 2 - \delta \Rightarrow |\!|\!|x - y |\!|\!| < \varepsilon . \end{aligned}$$

### Corollary 5.2

Suppose $(y_n)_{n=1}^\infty$ is relatively weakly compact in $L_1[0,1]$ and satisfies

5.1 $$\begin{aligned} \lim _{m,n \rightarrow \infty }[ |\!|\!|y_n + y_m |\!|\!|^2- 2(|\!|\!|y_n|\!|\!|^2 + |\!|\!|y_m|\!|\!|^2)] = 0. \end{aligned}$$

Then $(y_n)_{n=1}^\infty$ converges in $L_1[0,1]$.

### Proof

The proof is omitted as it is essentially the same as the proof that a uniformly convex norm is 2*R*. $\square$

### Theorem 5.3

Let $(Y,|\cdot |)$ be a rearrangement-invariant space on [0, 1]. Then *Y* admits an equivalent rearrangement-invariant norm $\Vert \cdot \Vert$ such that if $(y_n)_{n=1}^\infty$ is relatively weakly compact in *Y* and satisfies

5.2 $$\begin{aligned} \lim _{m,n \rightarrow \infty }[ \Vert y_m + y_n\Vert ^2 - 2(\Vert y_m\Vert ^2 + \Vert y_n\Vert ^2) ]= 0, \end{aligned}$$

then $(y_n)_{n-1}^\infty$ converges weakly in *Y*. In particular, $\Vert \cdot \Vert$ restricts to a *W*2*R* norm on every reflexive subspace of *Y*.

### Proof

Note that *Y* embeds continuously into $L_1[0,1]$. Define $\Vert \cdot \Vert$ as follows:

$$\begin{aligned} \Vert y\Vert = (|y|^2 + |\!|\!|y|\!|\!|^2)^{1/2} \quad (y \in Y). \end{aligned}$$

Suppose that $(y_n)_{n=1}^\infty$ satisfies (5.2). Then $(y_n)_{n=1}^\infty$ also satisfies (5.1) and $(y_n)_{n=1}^\infty$ is relatively weakly compact in $L_1[0,1]$. It follows from Theorem D that $(y_n)_{n=1}^\infty$ converges in $L_1[0,1]$, which implies that $(y_n)_{n=1}^\infty$ has a unique weak cluster point in *Y*, i.e. that $(y_n)_{n=1}^\infty$ converges weakly in *Y*. $\square$

### Corollary 5.4

$L_\infty [0,1]$ admits an equivalent rearrangement-invariant norm which restricts to a *W*2*R* norm on every reflexive subspace.

## Data Availability

Not applicable.
